# Complete Genome Sequence of Staphylococcus aureus Siphophage Lorac

**DOI:** 10.1128/MRA.00586-19

**Published:** 2019-07-03

**Authors:** Antoine Marc, Katie Cater, Rohit Kongari, Asma Hatoum-Aslan, Ryland F. Young, Mei Liu

**Affiliations:** a Center for Phage Technology, Texas A&M University, College Station, Texas, USA; b Department of Biological Sciences, The University of Alabama, Tuscaloosa, Alabama, USA; Queens College

## Abstract

Staphylococcus aureus is a leading cause of a wide range of clinical infections. Here, we announce the complete genome sequence of S. aureus siphophage Lorac, a phiETA-like temperate phage that is similar at the nucleotide level to the previously described S. aureus prophage phiNM2.

## ANNOUNCEMENT

Staphylococcus aureus is a Gram-positive skin-associated bacterium and is the causative agent for a wide range of clinical infections, including bacteremia, infective endocarditis, and pleuropulmonary and other device-related infections (1). Temperate phages, such as phiETA, are able to move between S. aureus strains and can encode virulence factors or toxins (2).

Siphophage Lorac was isolated from the wastewater treatment plant in Tuscaloosa, Alabama, in August 2015 using the host strain S. aureus strain RN4220. Host bacteria were cultured on tryptic soy broth or agar (Difco) at 37°C with aeration. Phages were cultured and propagated by the soft agar overlay method (3). It was identified as a siphophage using negative-stain transmission electron microscopy performed at the University of Alabama Optical Analysis Facility, as described previously (4). Phage genomic DNA was prepared using a modified Promega Wizard DNA cleanup kit protocol as described previously (4). Pooled indexed DNA libraries were prepared using the Illumina TruSeq Nano low-throughput (LT) kit, and the sequence was obtained from the Illumina MiSeq platform using the MiSeq v2 500-cycle reagent kit following the manufacturer’s instructions, producing 631,646 reads for the index containing the phage genome. FastQC 0.11.5 (https://www.bioinformatics.babraham.ac.uk/projects/fastqc/) was used to quality control reads. The reads were trimmed with FastX-Toolkit 0.0.14 (http://hannonlab.cshl.edu/fastx_toolkit/download.html) before being assembled using SPAdes 3.5.0 (5). Contig completion was confirmed by PCR using primers (5′-GTCCCTATCAAACCGAGAATCC-3′ and 5′-ACATGGGTGTAATCGACAAAGA-3′) facing off the ends of the assembled contig and Sanger sequencing of the resulting product, with the contig sequence manually corrected to match the resulting Sanger sequencing read. GLIMMER 3.0 (6) and MetaGeneAnnotator 1.0 (7) were used to predict protein-coding genes with manual correction for appropriate gene starts, and tRNA genes were predicted with ARAGORN 2.36 (8). Rho-independent termination sites were identified via TransTerm (http://transterm.cbcb.umd.edu/). Sequence similarity searches were performed by BLASTp 2.2.28 (9) against the NCBI nonredundant (nr), UniProt Swiss-Prot (10), and TrEMBL databases. InterProScan 5.15-54.0 (11), LipoP (12), and TMHMM v2.0 (13) were used to predict protein function. All analyses were conducted at default settings via the CPT Galaxy (14) and WebApollo (15) interfaces (https://cpt.tamu.edu/galaxy-pub).

Lorac was assembled as a complete genome with 43,147 bp and 1,371-fold coverage. It has a G+C content of 34%, which is similar to that of its host (16). Lorac is probably a temperate phage since it belongs to the *Phietavirus* genus and is 99.18% similar at the nucleotide level to phiNM2 (GenBank accession no. DQ530360), a prophage identified in S. aureus strain Newman (17). Morphogenesis genes coding for the major capsid protein, scaffolding protein, TerL, TerS, tail proteins, tail-head connector protein, portal protein, tape measure protein, and tape measure chaperone were annotated. Lysogeny-associated proteins, including Cro-like and cI-like regulators, excisionase, and integrase, were identified, as was a lysis cassette consisting of a class II holin and an amidase-type endolysin.

### Data availability.

The genome sequence of phage Lorac was submitted to GenBank under accession no. MH321492. Associated BioProject, SRA, and BioSample accession numbers are PRJNA222858, SRR8788599, and SAMN11260821, respectively.
